# Rural Land Transfer and Urban Settlement Intentions of Rural Migrants: Evidence from a Rural Land System Reform in China

**DOI:** 10.3390/ijerph20042817

**Published:** 2023-02-05

**Authors:** Yinxin Su, Mingzhi Hu, Yuzhe Wu

**Affiliations:** 1 School of Public Affairs, Zhejiang University, Hangzhou 310027, China; 2 School of Management, Chinese Academy of Housing and Real Estate, Zhejiang University of Technology, Hangzhou 310023, China

**Keywords:** migrant, settlement intention, land transfer, land reform

## Abstract

Using data from the China Migrants Dynamic Survey, this paper provides new evidence on the impact of rural land transfer on urban settlement intentions of rural migrants. There was a rural land system reform in rural China that provided increased compensation for rural land expropriation and allowed the transaction of collective construction land for business purposes. We determine an increase in urban settlement intentions of rural migrants following the reform as an exogenous change in rural land transfer of rural migrants. We examine two mechanisms that may explain how the reform increased the settlement intentions of rural migrants, and our empirical evidence suggests that the reform increased social integration and reduced rural place attachment of rural migrants. Furthermore, we determine variations in the effect of the reform across migrants of various ages, social security benefits, and migration distances. Overall, this study extends the implications of the market-oriented rural land reform to sustainable and inclusive urbanization and highlights the role of social integration and rural place attachment in migration decisions.

## 1. Introduction

China has undergone rapid urbanization since the late 1980s [1]. Statistics from China’s National Bureau of Statistics indicate that the urbanization rate in China has grown rapidly from 17.9% in 1978 to 64.72% in 2021. Massive rural-to-urban migration has flooded most Chinese cities as regulations on internal migration have been slowly relaxed [2]. The number of migrants in China has increased from 6.57 million in 1982 to 281.7 million in 2016 [3]. The rapid urbanization and large scale of rural-to-urban migration has caused serious problems, such as rural decline and farmland abandonment [4,5,6,7,8,9,10]. Land ownership and land use rights are separated under the current rural land system: land is owned by the village collective, while individual villagers only have land use rights. Villagers have no right to sell their contracted land even though they have actually settled in cities and permanently left the countryside. The land system leads to rising rural labor migration costs and widespread farmland abandonment [11,12,13,14,15,16].

Rural land is considered the rural migrants’ means of social security or insurance to combat unexpected risks in the city, such as unemployment and illness [17]. Rural migrants are less likely to cede land use rights in the countryside, even though the land has been actually abandoned [7,18,19]. To promote market-oriented allocation of land resources, the Chinese government implemented a rural land system reform that provided increased compensation for rural land expropriation and allowed the transaction of collective construction land for business purposes from early 2015 to late 2017, with 33 counties (cities and districts) selected as the pilot sites [20] (In February 2015, the 13th meeting of the 12th session of the National People’s Congress passed the *Decision on Authorizing State Council to Temporarily Adjust the Implementation of Relevant Laws and Rules in Administrative Regions of 33 Pilot Counties (Cites, Districts) Like Daxing District, etc. of Beijing*, and decided to temporarily stop implementing the provisions on not allowing the transfer of collective construction land usage right in Article 43 and Article 63 of the *Land Administration Law* and Article 9 of the *Law on Administration of Urban Real Estates*. The 33 pilot sites include Daxing District of Beijing city, Ji County of Tianjin city, Dingzhou city of Hebei Province, Zezhou County of Shanxi Province, and Horinger County of Inner Mongolia, Haicheng County of Liaoning Province, Jiutai District of Changchun city, Anda city of Heilongjiang Province, Songjiang District of Shanghai city, Wujin District of Changzhou city, Yiwu city of Zhejiang Province, Deqing County of Zhejiang Province, Jinzhai County of Anhui Province, Jinjiang city of Fujian Province, Yujiang County of Jiangxi Province, Yucheng city of Shandong Province, Changyuan County of Henan Province, Yicheng city of Hubei Province, Liuyang city of Hunan Province, Nanhai District of Foshan city, Beiliu city of Guangxi Province, Wenchang city of Hainan Province, Dazu District of Chongqing city, Pi County of Sichuan Province, Lu County of Sichuan Province, Meitan County of Guizhou Province, Dali city of Yunnan Province, Qushui County of Tibet, Gaoling District of Xi’an city, Longxi County of Gansu Province, Huangyuan County of Qinghai Province, Pingluo County of Ningxia Province, and Yining City of Xinjiang Province). The land reform promotes the transaction of land use rights in rural areas by increasing potential benefits from rural land transfers.

Using data from the China Migrants Dynamic Survey, this paper examines the impact of rural land transfer on urban settlement intentions of rural migrants. By exploiting a rural land system reform in rural China that provided increased compensation for rural land expropriation and allowed the transaction of collective construction land for business purposes as an exogenous change in rural land transfer of rural migrants, we find an increase in the urban settlement intentions of rural migrants following the reform. In addition, we examine two mechanisms that might explain how the reform increased settlement intentions of rural migrants. The results suggest that the reform increased social integration and reduced rural place attachment of rural migrants. Furthermore, we find variation in the effect of the reform across migrants of different ages, social security benefits, and migration distances.

This paper contributes to the literature on the effect of land rights on household decision-making in several ways. First, previous studies have primarily focused on the effects of individual characteristics and factors in destination cities on the urban settlement intentions of rural migrants [21,22,23,24,25,26]. Less attention has been paid to the barriers to migration out of rural areas. This paper examines how the urban settlement intentions of rural migrants are associated with rural land transfer. Several studies have examined the effect of rural landholdings on rural–urban migration in China, and the results are inconsistent. On the one hand, a few investigations indicate a positive relationship between rural landholdings and participation in outside labor markets due to the wealth effect of the land resources in rural China. Using data from a migrant survey in Jiangsu Province, Hao and Tang (2015) revealed that the possession of farmland or housing land in rural areas significantly decreases the intention of rural migrants to obtain an urban hukou in their destination cities [17]. Using data from a rural household survey conducted in Sichuan Province, Zhao (1999) determined that a decrease in the size of household land increases the probability of migration because the shortage of farmland reduces the relative marginal income from labor in farming [27]. Mullan et al. (2011) also observed that in the absence of complete property rights, increased land tenure security reduces the likelihood of migration [14]. On the other hand, rural landholdings are suggested to promote migration by several studies. Using data from three villages in the northeast of the Jiangxi Province, Feng and Heerink (2008) have shown that households with smaller landholdings are less likely to migrate due to economic constraints [12]. Rural land is also determined to encourage return-migration because it is an incentive for rural migrants to maintain strong ties with the village [28,29]. Some studies indicate an insignificant relationship between rural land and migration. For example, using data from the Chinese Household Income Project, Li and Zahniser (2002) determined that the amount of land controlled by a household on average does not have a significant effect on migration, although increased farming income reduces the probability of migration [19]. The inconsistent results in previous analyses may be driven by the endogeneity issue of land transfer choice. For example, rural migrants who have rural landholdings tend to have a higher likelihood of return migration and a lower probability of urban settlement intention. The research design adopted in this paper, which exploits the rural land system reform in rural China as an exogenous change in rural land transfer of rural migrants, helps mitigate this concern.

The second key contribution of this paper aims to shed light on the mechanisms behind the relationship between rural land transfer and settlement intentions. Using data from the China Migrants Dynamic Survey (CMDS), this paper provides new evidence on the rural land transfer’s impact on the urban settlement intentions of rural migrants. Our results indicate that an increase in the urban settlement intention of rural migrants following the reform that provided increased compensation for rural land expropriation and allowed the transaction of collective construction land for business purposes. We examine two mechanisms that may explain how the reform increased the settlement intentions of rural migrants, and our empirical evidence suggests that the reform increases the social integration of rural migrants into the urban life and reduces their rural place attachment. The examination of the potential mechanisms helps in obtaining a better understanding of the effects of rural landholdings on the urban settlement intention or return migration of rural migrants. We determine variations in the effect of the reform across migrants of various ages, social security benefits, and migration distances, which provides supportive evidence to the baseline results and carries broad policy implications.

The rest of this paper is structured as follows. Section 2 introduces the background of China’s rural land system reform and develops the key hypothesis that the reform affects the settlement intentions of rural migrants. Section 3 describes our data and empirical specification. Section 4 reports the results from baseline regression and several robustness checks. Section 5 investigates the heterogeneity effect of the reform. Section 6 concludes this study with some brief remarks.

## 2. Research Background and Hypothesis Development

### 2.1. China’s Rural Land System Reform

China has a dual land ownership system, wherein rural land is owned by village collectives and urban land is publicly owned by the state since the founding of the People’s Republic of China in 1949. There are huge conflicts of interest between individual land users, local collectives, and the state [30,31]. First of all, land belonging to rural collectives can be only transferred within the collective’s members; local collectives do not have the right to convert the land use, such as conversion from agricultural use to real estate development. Realizing that the collective rural land system increases conflicts between the efficient use of rural land and its retention as security for the rural population [32], the Household Responsibility System, introduced around 1980, has individualized residual income and some management rights to agricultural land [30], while the Rural Land Contract Law, passed in 2002, further ensued the long-term stability of the relationship of land contract in rural areas. Villagers who have rural land rights obtain higher benefits due to a longer contract duration, reduced frequency of land reallocations, and increased flexibility of subcontracting farmland to fellow villagers and leasing land to external investors after these policies are implemented [14,17].

However, the nature of ownership of the contracted land remains unchanged. The collective ownership of rural land makes it impossible for the villagers to enjoy the land-value-added income by land use conversion such as from agricultural use to real estate development [32]. Due to soaring housing prices after the housing reform in the early 1990s that allowed public housing tenants to buy their state-owned housing units at heavily subsidized prices [33], local governments have been incentivized to obtain land from the rural collectives at low prices through an expropriation process [34]. Urbanization has been increasingly implemented on the local scale through rural land acquisition that finances urban infrastructure construction [35,36]. The massive rural-out migration has dramatically affected rural land use in China, and many serious related problems have arisen, such as farmland abandonment [6,7].

To improve the rural land system and promote sustainable urbanization, the Chinese government implemented a rural land system reform in early 2015, with 33 counties (cities and districts) selected as the pilot sites [20]. The reform ended at the end of 2017. During the reform period, the usage right of rural collectively owned profit-oriented construction lands in stock was allowed to be transferred, leased and converted into shares in these pilot sites [20]. The usage rights of rural collective profit-making construction lands execute the same entry-into-market function, same right and same price as the usage rights of state-owned construction lands. The farmers whose land has been expropriated by the local government in the pilot areas can enjoy a higher share in the land-value-added benefits than their counterparts in other areas. Rural migrants coming from these pilot areas are probably more likely to transfer their land rights in rural areas due to the increased compensation for rural land expropriation and approved transaction of collective construction land for business purposes.

### 2.2. The Rural Land System Reform and Settlement Intentions of Rural Migrants

Rural land has huge implications on the migration and urban settlement intentions of the rural population. The neoclassical economics and the new economics of labor migration theory consider migration as a cost–benefit decision based on the maximization of individual utility [37]. Settlement intention can be affected by push factors at the origin and pull factors at the destination, that is, the positives of staying and the negatives of moving, as well as the opposite [38]. The rural land system reform in China may increase the urban settlement intentions of rural migrants for several reasons.

First, the rural land system reform would increase the probabilities of ceding the land use rights in the countryside for rural migrants because it greatly increases land transfer revenue. Rural land plays an important role for rural villagers as a form of social security, which allows rural migrants to survive potentially vulnerable situations, such as illness and unemployment [39]. Many rural migrants regularly return to their villages and retain their ties to the land as part of a strategy of spatial and sectoral diversification of household labor [28]. For rural migrants who work and live in cities and still have land use rights in their home villages, the rural land that represents security and links to the village may stimulate return migration [40]. After relinquishing their rural lands, rural migrants not only lose their potential rural income, but also the security of land tenure and place attachment. Weak social networks and social interaction in the origin place may decrease the probability of return migration [41,42].

Second, the rural land system reform would increase the social integration of rural migrants in urban life. The pilot areas during the reform period tried to establish a rural land transaction market by allowing the transfer of rural construction land use rights on the basis of retaining ownership as a collective [40]. The compensation for land expropriation in the pilot areas is higher than the statutory standards [20]. The transfer of rural land use rights in the pilot areas provide considerable wealth through land expropriation or market transactions. With the money obtained from the transfer of rural land contractual operation rights, rural migrants could integrate into urban life better. For example, housing has become much more expensive in Chinese cities with prices soaring in the last decade [43]. Purchasing an urban home with rural land monetary compensation could be easier. Homeowners have been found to be more likely to participate in local activities, and thus, have more social networks and resources [44,45,46,47]. Social attachment and interaction with the destination may encourage the full integration and permanent settlement of rural migrants in cities [48].

## 3. Data and Identification Strategy

### 3.1. Data Source

Our analysis is based on the constructed dataset from the China Migrants Dynamic Survey (CMDS), which is a nationwide household survey conducted by the Migrant Population Service Center at the National Health Commission of China. CMDS focuses on the migrant population who are aged 15 or above, have a non-local hukou and have resided in their current city for one month and longer. A stratified and multi-stage sampling with probability proportional to size is used in this survey for a better representation of Chinese society. The first wave of the CMDS survey began in 2010, and was conducted annually afterwards. The latest available data of this survey were gathered in 2018. We used data from the 2017 wave, as only this wave comprises information on the hometown of migrants after the land reform in 2015. Approximately 169,989 migrants working or living in 31 mainland provinces or their administrative equivalents (municipalities and autonomous regions) in China were interviewed in the 2017 CMDS.

The CMDS dataset is unique for the current study in several respects. First, the CMDS incorporates the hukou registered residence (district or county) of each respondent, from which we can define whether the respondent’s hometown is one of the pilot areas during the rural land system reform. Second, respondents are asked if they have decided to stay in their current city. Based on the answer to this question, we can measure the respondents’ settlement intentions. Third, the dataset comprises detailed household characteristics (i.e., age, gender, health status, medical insurance, housing tenure, educational attainment, marital status, household income, and migration distance), and socioeconomic characteristics (i.e., education, self-employment, household wealth, and household income). These details allow us to control not only for the common variables (e.g., age, gender, health status, housing tenure, educational attainment, marital status, and household income) that are found to be critical in explaining settlement intentions, but also for several unique social and cultural variables (e.g., medical insurance and migration distance).

### 3.2. Identification Strategy

The regressions with a binary outcome of settlement intentions in this analysis are estimated with a logistic regression. The baseline model that examines the relationship between the rural land system reform and settlement intentions of rural migrants is implemented in the following form:

(1) $$Settlement\;intention_{ij}=\beta_{0}+\beta_{1}Treatment_{ij}+X_{ij}+\theta_{j}+\varepsilon_{ij}$$

where the dependent variable $Settlement\;intention_{ij}$ is an indicator variable of settlement intention that equals 1 if the respondent *i* in province *j* has decided to stay in their current city and 0 if otherwise. $Treatment_{ij}$ is an indicator variable of the treatment group that is equivalent to 1 if the respondent’s hometown or the city that governs the hometown is one if the pilot areas during the rural land system reform and 0 if the respondent’s hometown or the city that governs the hometown is not. $X_{ij}$ is a vector of household characteristics, as described in Table 1. Province dummies ($\theta_{j}$) are included to control for time-invariant characteristics at the province level. Finally, $\varepsilon_{ijt}$ is the error term.

We apply two screenings to the sample. First, we restrict our attention to the migrants whose hometown and the city that governs the hometown was designated as a pilot area during the land reform. This allows individuals in the treatment and control groups to be comparable. Second, we eliminate observations with missing values. The final sample contains a total of 11,802 observations from 31 provinces in mainland China, of which 1459 respondents migrated from the pilot areas of the land reform (treatment group), while the remaining 10,343 are those who migrate from non-pilot areas (control group).

Table 1 provides summary statistics for the full sample and two subsamples of treatment and control groups. In the dataset, 80.99% of respondents have decided to stay in their current city. Migrants who are affected by the land reform and those who are not have shown different settlement intentions. Approximately 82.19% of respondents in the treatment group and 80.83% in the control group are likely to settle down in cities. The average age of the respondents in our sample is around 37; 57.03% of respondents are men; 83.07% have a good health status; 5.61% are communists; 42.25% have medical insurance; 20.50% have a college degree or higher. Overall, the average annual personal income is 4406 yuan per month. Only 14.49% of respondents migrate from the rural areas of the destination city, indicating a long-distance migration on average for the sample population of rural migrants. Overall, Table 1 reveals that migrants affected by the land reform are more likely to stay in their current city than those unaffected, unconditionally. The treatment group differs from the control group along several dimensions. These differences highlight that further investigation by controlling for these characteristics is needed.

## 4. Empirical Results

### 4.1. Baseline Regression Results

We examine how the land reform affects the settlement intentions of rural migrants based on Equation (1). The estimated coefficients, standard errors, significance levels and marginal effects are reported in Table 2. Column (1) of Table 2 shows the simplest specification by controlling for the treatment effect and province dummies only. Results indicate that, without controlling for household characteristics, the land reform significantly increases the probability of migrants settling down in the city which they have moved to by 2.84 percentage points for the treatment group relative to the control group. Column (2) of Table 2 further controls for the household characteristics, while the results consistently show a significant and positive effect of the land reform on the rural migrants’ urban settlement intentions.

Coefficients of the additional control variables in the model are as expected. For example, an inverse U-shaped relationship exists between the age of rural migrants and their settlement intentions. Migrants who are healthier, married, and highly educated are more likely to stay in cities. Homeownership increases the probability of migrants settling down in cities. Social security benefits provided in cities could increase the willingness of the floating population to stay because they help mitigate the impact of negative shocks in urban life. We determine that rural migrants who have urban medical insurance are more likely to settle down in cities. Another interesting find is that short migration (i.e., intra-city migration) is associated with a higher likelihood of settling down in cities compared with long migration (i.e., inter-city migration).

### 4.2. Robustness Checks

#### 4.2.1. Unobservable Megacity Characteristics

Migrants, especially those who have arrived relatively recently, tend to cluster in large cities, as large cities have better job opportunities, infrastructure, and social welfare than other cities [49]. Rural areas of large cities are more desirable and habitable because large cities can afford greater investments in their rural areas than other cities. Migrants who work or live in large cities are more likely to settle down, and those who migrate from rural areas of large cities are more likely to move back to their hometowns. Thus, the baseline findings in Table 1 are threatened by a potential issue—the self-selection bias. Rural migrants who are more likely to settle down in cities (or move back to their hometowns) tend to move into large cities (or come from rural areas of large cities).

To correct this potential self-selection bias, we exclude two main types of large cities in China: municipalities and province capitals. Column (1) of Table 3 reported the results by excluding instances of responders who migrated from rural areas of municipalities, and in Column (2) of Table 3 further we deleted instances of responders who migrated from province capitals. In Columns (3) of Table 3, we reported the results by excluding observations in municipalities, and in Column (4) of Table 3 we further deleted observations in provincial capitals. The coefficient of *Treatment* remains positive and significant at least at the 5% level throughout Columns (1) to (4) of Table 3. Therefore, self-selection of urban areas of large cities or of rural areas of large cities pose little threat to our baseline results.

#### 4.2.2. Controlling for Model Misspecification

Our previous estimations assume linear impacts of the land reform and control variables on the urban settlement intentions of rural migrants. If this model assumption is invalid, our previous estimations may be biased due to functional misspecification. We apply the propensity score matching (PSM) approach, the identification of which compares treatment and control units with similar values on the propensity score (i.e., the conditional probability of being treated given a set of covariates) to deal with this potential issue [50]. The estimation results of the PSM approach are reported in Table 4. Consistent with the baseline results, we determine that rural migrants in the treatment group are 3.3 percentage points more likely to settle down in cities, and the difference is significant at the 5% level. Thus, the baseline results are robust to model misspecification.

**Figure 1:**
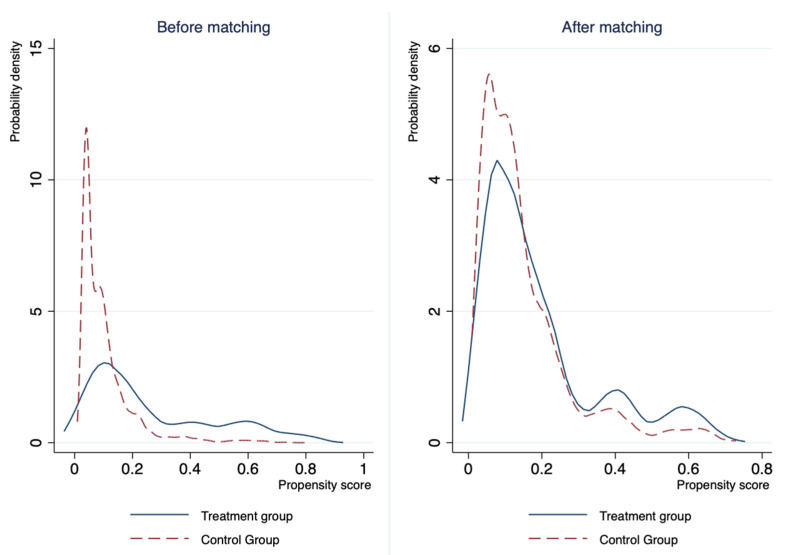
The distribution of propensity scores.

The density distribution of the propensity score between the treatment and control groups before and after PSM is depicted in Figure 1. The solid curves denote the possibility of migrating from the pilot areas during the land reform for the migrants in the treatment group, which is estimated by a logistic regression. The dashed line curves denote the possibility of migrating from the pilot areas during the land reform for the migrants in the control group, which is estimated by a logistic regression. The PSM has eliminated the significant distribution differences that existed before PSM, indicating that our PSM estimation is reliable.

#### 4.2.3. Falsification Test

We conducted a falsification test in which we randomly assigned the treatment effect to the migrants. As there are 1459 migrants in the treatment group, we randomly chose 1459 migrants in the sample and considered them as migrants from the pilot areas of the land reform, which comprises the pseudo treatment group. We ran the baseline regression model (i.e., Equation (1)) with the random assignments 30 times to obtain consistent results. Results are estimated with logistic regressions and reported in Table 5. Our baseline results are supported by the following evidence: First, only in two assignments can we observe a significant coefficient of treatment, as shown in Columns (17) and (28). Second, with respect to the two cases containing significant coefficients of treatment, the signs of the coefficient are negative. This wrongly indicates that migrants in the treatment group are less likely to settle down. These findings provide supportive evidence that our baseline results are not spurious.

### 4.3. Possible Mechanisms

There are two possible mechanisms that the land reform has a positive effect on the settlement intentions of rural migrants. First, we attempted to examine whether the land reform promotes the social integration of rural migrants in cities. We conceptualized social integration as ties with the natives, which is similar to Toruńczyk-Ruiz and Brunarska (2020) [48]. Migrant populations tend to spend more time with people coming from the same rural areas as themselves. The lack of communication with local people reflects the poor integration into their current city. In the 2017 CMDS, each respondent was asked who he/she socializes with most in his/her spare time. The choices for this question were as follows: (1) Migrants from your hometown; (2) Migrants from somewhere except for your hometown; (3) Local people; and (4) Rarely communicate with others. Our data shows that 21.17% of migrants in the sample rarely communicate with others in their spare time, 31.53% of them tend to communicate most with other migrants from their same hometowns, and only 36.77% of migrants spend most time with locals. We constructed an indicator variable of social integration that is equivalent to 1 if respondents socialize most with locals in their spare time and 0 if otherwise, which is used as a proxy for the social integration of rural migrants into urban life.

We replaced the dependent variable in the baseline model, i.e., Equation (1), with the indicator variable of social integration, and the estimated results are reported in Column (1) of Table 6, which examines the relationship between land reform and the social integration of rural migrants. As expected, we determined that the land reform significantly promotes the social integration of rural migrants. Compared with migrants from the control group, those from the treatment group are 4.22 percentage points more likely to communicate with local people.

Another possibility is that the land reform reduces the place attachment of rural migrants to their hometowns. The respondents in the survey were queried about whether they have contracted land in their hometowns. We define an indicator variable of rural place attachment as being equal to 1 if the respondent has contracted land in his/her hometown and being equal to 0 if otherwise based on this information. Column (2) of Table 6 reports the regression results by replacing the dependent variable in the baseline model with the indicator variable of rural place attachment. Results show that migrants in the treatment group are more likely to lose their contracted land in their rural hometowns than those in the control group, indicating that land reform decreases the rural migrants’ attachment to their hometowns.

## 5. Further Analyses

We investigate the heterogeneity effect of the land reform on the settlement intentions of migrants with different characteristics in this section. This attempt helps enrich our understanding of how the land reform affects the settlement choices of rural migrants.

First, we classify migrants into two categories according to their age: young (age 40 and below) and old migrants (age 41 and above). We focus on the age difference because old migrants are more willing to move back to their hometowns than young migrants, and thus, could be less vulnerable to the effect of the land reform. Results in Column (1) of Table 7 show that the land reform significantly increases the settlement intentions of young migrants, while its impact on old migrants is insignificant.

Second, we examined whether the effect of the land reform varies between migrants who have urban medical insurance and those who do not have urban medical insurance. We replaced the dependent variable in the baseline model with the interaction terms between an indicator variable of the treatment group, an indicator variable of having urban medical insurance, and an indicator variable of having no urban medical insurance to test this hypothesis. The results, as reported in Column (2) of Table 7, show that land reform has a significant and positive effect on the settlement intention of migrants who have urban medical insurance, while its effect is insignificant for migrants who do not have urban medical insurance.

Third, we investigated the cross effect of the land reform and migration distance on settlement intentions of migrants by replacing the dependent variable in the baseline model with the following two variables. The first one is the interaction term between an indicator variable of the treatment group and an indicator of intra-city migration. The second one is the interaction term between an indicator variable of the treatment group and an indicator of inter-city migration that equals 1 if the respondent migrates from rural areas of other cities and equals 0 if otherwise. The regression results are reported in Column (3) of Table 7. The land reform significantly increases the settlement intentions of migrants who come from the rural areas of the destination city, while its influence on the migrants who come from the rural areas of other cities is not obvious.

## 6. Conclusions

This paper examines the rural land transfer’s effect on the urban settlement intentions of rural migrants by using a land reform that increases the probability that farmers give up their right to use rural land. It determines that the land reform encourages the urban settlements intentions and this impact varies across migrants of different ages, social security benefits, and migration distances. This study identifies a relatively novel source of diversity in out-migration among rural–urban migrants. The study extends the implications of the market-oriented rural land reform to sustainable and inclusive urbanization and highlights the role of social integration and rural place attachment in migration decisions.

Temporary migration, or circular migration, is a predominant pattern form of domestic labor mobility in many developing countries [27,28,29,48], and it prevails further in China due to the present household registration system [51]. Temporary migrants are those who regularly return to their villages during the Spring Festival and the planting and harvesting seasons, and retain their ties to the land as part of a strategy of spatial and sectoral diversification of household labor. Rural housing built by the temporary migrants has resulted excessive expenditure, room vacancy, village-hollowing, and loss of arable land, as well as decreasing the potential of ensuring food security in many agricultural villages [52,53,54]. The large-scale temporary migration greatly contributes to the industrialization and urbanization in China’s post-reform development by providing cheap and flexible labor forces, but it has been increasingly regarded as unsustainable for the future [24,55].

Recent research has gradually shifted from examining motives for migration in general to motives for permanent migration [51]. Permanent migrants in China are usually defined as those who have converted their rural hukou to urban hukou [24,51] because the conversion from rural to urban hukou requires the relinquishment of rural land due to China’s hukou registration system [17]. The desire for or resistance to the hukou conversion is essentially a trade-off between rural and urban benefits. This paper contributes to an emergent but rapidly growing literature concerned with the urban settlement intentions of rural migrants. Settlement intention is the intention to settle in cities permanently, as opposed to returning to the countryside. Understanding the determinants of urban settlement intentions is important to predict future migration flows and trends. It helps formulate policies that increase the desire to settle in cities and reduce temporary migration. Our results show that rural land transfer promotes settlement intentions in destination cities. This indicates that the reluctance to cede rural land for no or unfair payment is an important reason for the unwillingness of hukou conversion and settlement in destination cities. The hukou registration system and incomplete rural property rights have made urbanization in China a much slower process than it would otherwise have been.

The results are subjected to some limitations that outline an agenda for future research. We need to emphasize that the two transmission mechanisms we identified may only contribute to partial effects, indicating that the land reform may influence settlement intentions through other channels. We are aware that the proxy variables we used are far from perfect. The proxy variable of social integration is constructed based on the subjective survey question that “who do you socialize with most in your spare time”, and the rural place attachment is measured by the question “whether you have contracted land in your hometowns.” Future studies can use alternative measurements with additional data to measure social integration and place attachment. We do not consider the moderating effect of institutional factors and city characteristics. For example, rural migrants are often discriminated against in the urban labor market and access to social benefits and public services [56]. Further research could test whether the effect of the land reform varies across cities with various levels of discrimination against rural migrants.

## Figures and Tables

**Table 1:** Summary statistics.

	Full Sample		Treatment Group		Control Group	
	Mean	Std.Dev.	Mean	Std.Dev.	Mean	Std.Dev.
Settlement intention	0.8099	0.3924	0.8218	0.3828	0.8083	0.3937
Treatment	0.1236	0.3292				
Age	36.729	10.304	35.451	9.890	36.909	10.349
Male	0.5703	0.4951	0.5579	0.4968	0.5721	0.4948
Health status	0.8307	0.3750	0.8369	0.3696	0.8298	0.3758
Communist	0.0561	0.2301	0.0583	0.2343	0.0558	0.2295
Insurance	0.4225	0.4940	0.3626	0.4809	0.4309	0.4952
Homeowner	0.2858	0.4518	0.2666	0.4423	0.2885	0.4531
College or above	0.2050	0.4037	0.2084	0.4063	0.2045	0.4033
Middle & high school	0.6218	0.4850	0.6669	0.4715	0.6154	0.4865
Married	0.7837	0.4118	0.7615	0.4263	0.7868	0.4096
Income	4,405.6	3,886.3	4,182.3	3,537.5	4,437.1	3,932.2
Intra-city migration	0.1449	0.352	0.1974	0.3982	0.1375	0.3444
Obserbations	11,802		1459		10,343	

Note: *Settlement intention* is an indicator variable that equals 1 if the respondent has decided to stay in their current city and 0 if otherwise. *Treatment* is an indicator variable that equals 1 if the respondent’s hometown or the city that governs the hometown is one of the pilot areas during the rural land system reform and 0 if otherwise. *Age* is the age of respondent. *Male* is an indicator variable of male respondent. *Health status* is an indicator variable that equals 1 if the respondent reports a good health status and 0 if otherwise. *Communist* is an indicator of members of the Communist Party of China. *Insurance* is an indicator variable of medical insurance that equals 1 if the respondent has urban employee’s medical insurance, urban resident’s medical insurance, or urban and rural resident’s cooperative medical insurance and 0 if otherwise. *Homeowner* is an indicator variable of homeownership that equals 1 if the respondent own a home and 0 if otherwise. *College or above* is an indicator variable that equals 1 if the respondent has a college degree or higher and 0 if otherwise. *Middle and high school* is an indicator variable that equals 1 if the highest education of the respondent is middle and high school and 0 if otherwise. *Married* is an indicator variable of being married. *Income* refers to the personal income measured as yuan per month. *Intra*-*city migration* is an indicator variable that equals 1 if the respondent migrates from rural areas of their current city and equals 0 if otherwise. Data source comes from CMDS 2017.

**Table 2:** Baseline regression results: impacts of the reform on settlement intentions.

	(1)		(2)	
	Coef.	Marginal Effect	Coef.	Marginal Effect
Treatment	0.1882 **	0.0284 **	0.1798 **	0.0265 **
	(0.0813)	(0.0123)	(0.0819)	(0.0120)
Age			0.0371 **	0.0055 **
			(0.0156)	(0.0023)
Age squared			−0.0005 ***	−0.0001 ***
			(0.0002)	(0.0000)
Male			0.0039	0.0006
			(0.0501)	(0.0074)
Health status			0.2173 ***	0.0320 ***
			(0.0640)	(0.0094)
Communist			−0.0737	−0.0109
			(0.1180)	(0.0174)
Insurance			0.2133 ***	0.0314 ***
			(0.0589)	(0.0087)
Homeowner			0.6597 ***	0.0971 ***
			(0.0651)	(0.0095)
College or above			0.4766 ***	0.0701 ***
			(0.0988)	(0.0145)
Middle and high school			0.2996 ***	0.0441 ***
			(0.0656)	(0.0096)
Married			0.2439 ***	0.0359 ***
			(0.0672)	(0.0099)
Income			0.0567 ***	0.0083 ***
			(0.0216)	(0.0032)
Intra-city migration			0.2179 **	0.0321 **
			(0.0906)	(0.0133)
Other controls				
Province dummies	Yes		Yes	
R-squared	0.0213		0.0464	
Observations	11,802		11,802	

Note: The dependent variable in this table is an indicator variable of settlement intention that equals 1 if the respondent has decided to stay in their current city and 0 if otherwise. Robust standard errors are reported in parentheses. ** and *** indicate significance at the 5%, and 1% levels, respectively. Local housing prices might play a pushing factor for rural migrants. We also have tried to control the log value of city-level housing prices in the model, and the results suggest that local housing prices are positively associated with rural migrants’ urban settlement intentions. The coefficient of “Treatment” remains positive and statistically significant at the 5% level after introducing housing price as an explanatory variable (coefficient = 0.2508, *p*-value = 0.0110).

**Table 3:** Robustness checks: excluding the effects of large cities.

	(1)		(2)	
	Exclusion of Original Cities Being Municipalities		Exclusion of Original Cities Being Municipalities and Province Capitals	
	Coef.	Marginal Effect	Coef.	Marginal Effect
Treatment	0.2309 **	0.0329 **	0.2504 **	0.0346 **
	(0.1046)	(0.0149)	(0.1116)	(0.0154)
Other controls				
Household characteristics	Yes		Yes	
Province dummies	Yes		Yes	
R-squared	0.0455		0.0649	
Observations	6494		4489	
	(3)		(4)	
	Exclusion of current cities being municipalities		Exclusion of current cities being municipalities and province capitals	
	Coef.	Marginal effect	Coef.	Marginal effect
Treatment	0.2294 ***	0.0369 ***	0.2790 ***	0.0456 ***
	(0.0875)	(0.0141)	(0.0951)	(0.0155)
Other controls				
Household characteristics	Yes		Yes	
Province dummies	Yes		Yes	
R-squared	0.0363		0.043	
Observations	8253		6972	

Note: The dependent variable in this table is an indicator variable of settlement intention that equals 1 if the respondent has decided to stay in their current city and 0 if otherwise. Robust standard errors are reported in parentheses. ** and *** indicate significance at the 5%, and 1% levels, respectively.

**Table 4:** Robustness check: PSM.

	(1)	
	Coef.	Marginal Effect
Treatment	0.222 **	0.033 **
	(0.095)	(0.014)
Other controls		
Household characteristics	Yes	
Province dummies	Yes	
R-squared	0.0683	
Observations	4105	

Note: The dependent variable in this table is an indicator variable of settlement intention that equals 1 if the respondent has decided to stay in their current city and 0 if otherwise. Robust standard errors are reported in parentheses. ** indicate significance at the 5% levels, respectively.

**Table 5:** Falsification test: random assignments of treatment.

	(1)	(2)	(3)	(4)	(5)	(6)	(7)	(8)	(9)	(10)
Treatment	−0.0131	0.0053	−0.0120	0.0279	0.0049	0.0026	−0.0462	−0.0451	−0.0985	0.0566
	(0.0721)	(0.0733)	(0.0723)	(0.0737)	(0.0732)	(0.0736)	(0.0722)	(0.0722)	(0.0718)	(0.0737)
Other controls										
Household characteristics	Yes	Yes	Yes	Yes	Yes	Yes	Yes	Yes	Yes	Yes
Province dummies	Yes	Yes	Yes	Yes	Yes	Yes	Yes	Yes	Yes	Yes
Observations	11,802	11,802	11,802	11,802	11,802	11,802	11,802	11,802	11,802	11,802
	(11)	(12)	(13)	(14)	(15)	(16)	(17)	(18)	(19)	(20)
Treatment	0.0297	0.0615	−0.0318	0.0109	−0.0133	−0.0892	−0.1617 **	0.0802	−0.0024	0.0872
	(0.0739)	(0.0741)	(0.0716)	(0.0735)	(0.0728)	(0.0720)	(0.0711)	(0.0743)	(0.0731)	(0.0744)
Other controls										
Household characteristics	Yes	Yes	Yes	Yes	Yes	Yes	Yes	Yes	Yes	Yes
Province dummies	Yes	Yes	Yes	Yes	Yes	Yes	Yes	Yes	Yes	Yes
Observations	11,802	11,802	11,802	11,802	11,802	11,802	11,802	11,802	11,802	11,802
	(21)	(22)	(23)	(24)	(25)	(26)	(27)	(28)	(29)	(30)
Treatment	0.0203	0.0057	0.0791	0.0529	−0.0470	0.0609	−0.0993	−0.1756 **	0.0009	−0.0627
	(0.0735)	(0.0736)	(0.0744)	(0.0744)	(0.0713)	(0.0746)	(0.0714)	(0.0700)	(0.0733)	(0.0723)
Other controls										
Household characteristics	Yes	Yes	Yes	Yes	Yes	Yes	Yes	Yes	Yes	Yes
Province dummies	Yes	Yes	Yes	Yes	Yes	Yes	Yes	Yes	Yes	Yes
Observations	11,802	11,802	11,802	11,802	11,802	11,802	11,802	11,802	11,802	11,802

Note: The dependent variable in this table is an indicator variable of settlement intention that equals 1 if the respondent has decided to stay in their current city and 0 if otherwise. Robust standard errors are reported in parentheses. ** indicate significance at the 5% levels, respectively.

**Table 6:** Possible mechanisms: social integration and rural place attachment.

	(1)		(2)	
	Coef.	Marginal Effect	Coef.	Marginal Effect
Treatment	0.2161 ***	0.0422 ***	−0.1522 **	-0.0289 **
	(0.0673)	(0.0131)	(0.0677)	(0.0128)
Other controls				
Household characteristics	Yes		Yes	
Province dummies	Yes		Yes	
R-squared	0.1322		0.0717	
Observations	11,802		11,802	

Note: The dependent variable in column (1) is an indicator variable of social integration that equals 1 if the respondent communicates more with locals than non-locals in his/her spare time and equals 0 if otherwise. The dependent variable in column (2) is an indicator variable of rural place attachment that equals 1 if the respondent has contracted land in his/her hometown and equals 0 if otherwise. The dependent variable in column (3) is an indicator variable of settlement intention that equals 1 if the respondent has decided to stay in their current city and 0 if otherwise. Robust standard errors are reported in parentheses. ** and *** indicate significance at the 5%, and 1% levels, respectively.

**Table 7:** Further analyses: heterogeneity results among individuals.

	(1)		(2)		(3)	
	Coef.	Marginal Effect	Coef.	Marginal Effect	Coef.	Marginal Effect
Treatment						
Old	0.1319	0.0194				
	(0.1304)	(0.0192)				
Young	0.2057 **	0.0303 **				
	(0.0974)	(0.0143)				
Treatment						
Insurance			0.4701 ***	0.0692 ***		
			(0.1538)	(0.0226)		
Noninsurance			0.0675	0.0099		
			(0.0935)	(0.0138)		
Treatment						
Intra-city migration					−0.0480	−0.0071
					(0.1892)	(0.0278)
Inter-city migration					0.2234 **	0.0329 **
					(0.0901)	(0.0132)
Other controls						
Household characteristics	Yes		Yes		Yes	
Province dummies	Yes		Yes		Yes	
R-squared	0.0464		0.0469		0.0466	
Observations	11,802		11,802		11,802	

Note: The dependent variable in this table is an indicator variable of settlement intention that equals 1 if the respondent has decided to stay in their current city and 0 if otherwise. Robust standard errors are reported in parentheses. ** and *** indicate significance at the 5%, and 1% levels, respectively. *Young migrants* is an indicator variable that equals 1 if the respondent’s age is 40 and below and 0 if otherwise. *Old migrants* is an indicator variable that equals 1 if the respondent’s age is above 40 and 0 if otherwise. *Insurance* is an indicator variable of medical insurance that equals 1 if the respondent has medical insurance and 0 if otherwise. *Noninsurance* is an indicator variable of medical insurance that equals 1 if the respondent does not have medical insurance and 0 if otherwise. *Intra-city migration* is an indicator variable that equals 1 if the respondent migrates from rural areas of their current city and equals 0 if otherwise. *Inter-city migration* is an indicator variable that equals 1 if the respondent migrates from rural areas of other cities and equals 0 if otherwise.

## Data Availability

Not applicable.
